# Evidence for the transformation of a subset of pulmonary carcinoids to small cell lung carcinomas through shared molecular alterations

**DOI:** 10.3389/fonc.2026.1768871

**Published:** 2026-03-20

**Authors:** Ruirui Fan, Jie Gao

**Affiliations:** 1 Department of Anatomia Pathology, The Islands Healthcare Complex-Macao Medical Center of Peking Union Medical College Hospital, Macao, Macao SAR, China; 2 Department of Pathology, Xiamen Humanity Hospital, Xiamen, China

**Keywords:** genetic analysis, neuroendocrine tumor, pathological analysis, pulmonary carcinoid, small cell lung carcinoma, tumor transformation

## Abstract

**Background:**

Pulmonary carcinoid and small cell lung carcinomas represent fundamentally distinct tumors in terms of molecular pathogenesis, pathological features, treatment, and prognosis. Whether any pathogenic link exists between pulmonary carcinoids and small cell carcinomas remains poorly understood. Elucidating the potential for carcinoids to undergo transformation into small cell lung carcinomas is critical for early intervention, treatment personalization, and prognostic assessment.

**Methods:**

H&E staining was performed to assess histomorphological transitions between pulmonary atypical carcinoids and small cell lung carcinomas. Immunohistochemistry was subsequently applied to compare the immunophenotypic profiles of two tumors. Molecular profiling was conducted to evaluate clonal relatedness and the presence of progressive genetic alterations, thereby providing evidence for transformation.

**Results:**

In addition to the atypical carcinoid and small cell lung carcinomas morphological characteristics, the lesion areas also present hybrid morphological characteristics of both tumors. Immunohistochemical analysis demonstrated distinct profiles between the two tumors: atypical carcinoids exhibited a low Ki67 proliferative index, high TP53 expression, and loss of RB1 expression, whereas small cell lung carcinomas showed a high Ki67 proliferative index, low TP53 expression, and loss of RB1 expression. Furthermore, clonal evolution analysis revealed that the two tumor components shared a set of 160 identical somatic mutations (accounting for approximately 16% of the total mutations in each component).

**Conclusion:**

This study delineates a dynamic evolutionary process within the pulmonary neuroendocrine tumor spectrum, confirming that a subset of atypical carcinoids can progress to small cell lung carcinoma through a transformation pathway.

## 1 Introduction

Lung neuroendocrine neoplasms (NENs) represent a heterogeneous group of neoplasms defined by distinct histopathological features and the expression of neuroendocrine immunohistochemical markers (1, 2). The pathological spectrum encompasses typical carcinoid (TC), atypical carcinoid (AC), small cell lung carcinoma (SCLC), and large cell neuroendocrine carcinoma (LCNEC). This classification framework, initially established in the 1999 WHO classification of lung tumors, has been endorsed by the European Neuroendocrine Tumor Society (ENETS) and further confirmed at a consensus meeting convened by the International Agency for Research on Cancer (IARC) (2). According to this current WHO-based classification, TC and AC are categorized as well-differentiated NETs corresponding to grade 1 (G1) and grade 2 (G2), respectively, whereas SCLC and LCNEC are classified as poorly differentiated neuroendocrine carcinomas (NECs) (3, 4). Notably, diffuse idiopathic pulmonary neuroendocrine cell hyperplasia (DIPNECH) as a precursor lesion of pulmonary NETs, along with relevant genetic syndromes, are also important components of the pulmonary neuroendocrine neoplasm spectrum associated with the development of neuroendocrine disorders (5). Current evidence indicates that the distinct pathogenic pathways of NETs and NECs underpin their differing epidemiology, biology, clinical management, and prognosis (6, 7). However, some studies suggest that these tumor types may harbor overlapping genetic alterations, albeit with divergent frequencies (8, 9). To date, research on tumor progression across this spectrum remains limited. Furthermore, solid evidence validating the transformation from NETs to NECs is still lacking. In recent years, the incidence of pulmonary carcinoid tumors has shown a consistent linear increase, albeit with a generally favorable prognosis (10, 11). In contrast, SCLC is characterized by aggressive biological behavior, including a propensity for early metastasis. No highly effective therapies currently exist to reliably control its progression. This therapeutic challenge directly contributes to its high malignancy and exceedingly poor prognosis. Carcinoids and SCLC exhibit marked disparities in their molecular profiles, expected survival, and treatment paradigms. Therefore, investigating the potential for carcinoid-to-SCLC transformation is of critical importance. Elucidating this process is vital for enabling early intervention, optimizing treatment approaches, and ultimately improving patient outcomes.

This study provides evidence that the pathogenesis of carcinoids and SCLC may overlap, and that at least a subset of carcinoids possesses the potential to transform into SCLC. This finding holds significant clinical implications, as it may allow for earlier intervention in carcinoid progression and offers new insights for personalized, precision therapy strategies. Among pulmonary NENs, the majority are NECs, accounting for approximately 95% of cases. SCLC represents the most common subtype (approximately 79%), while well-differentiated NETs comprise only about 5% (12, 13). NECs predominantly affect middle-aged and elderly males and demonstrate a strong association with smoking (14). In contrast, NETs occur more frequently in younger females and typically lack a clear smoking correlation (15). The advent of next-generation sequencing has profoundly advanced our understanding of the molecular epidemiology of cancer. Studies reveal that SCLC carries a high somatic tumor mutation burden (>7 mutations/Mb) (16). The co-mutation or loss of the tumor suppressor genes *TP53* and *RB1* represents a hallmark molecular alteration in SCLC pathogenesis (17). Early *TP53* inactivation drives genomic instability, leading to widespread allelic imbalances across multiple chromosomal regions. Concurrent *RB1* inactivation disrupts the *RB1/E2F* signaling pathway, a characteristic dysregulation in this malignancy. Other recurrent, though less frequent, molecular alterations include mutations in *MYCL, PTEN, STK11, EGFR, KRAS*, and *BRAF* (18–20). Overall, SCLCs are defined by pervasive chromosomal instability and extensive copy number variations. Consequently, these molecular features underpin their highly aggressive clinical course and poor prognosis.

Carcinoid are characterized by a low tumor mutational burden, with a somatic mutation rate typically below 1 mutation per megabase. The predominant genetic alterations affect chromatin remodeling genes, particularly components of the SWI/SNF complex, such as *MEN1, PSIP1, EIF1AX*, and *ARID1A* (21). Notably, these tumors generally lack mutations in key driver genes including *TP53, RB1, KRAS*, and *STK11/KEAP1 (*22). Researchers predominantly view carcinoids as a distinct tumor entity driven by an independent pathogenic mechanism. This perspective contrasts with their classification as an early developmental stage of SCLC. Advancements in diagnostic techniques and the implementation of early clinical interventions have enabled more precise monitoring. Consequently, reports of “histologic transformation” during treatment have become increasingly frequent. Studies indicate that approximately 3~10% of EGFR-mutant NSCLCs undergo transformation into SCLC. However, the underlying molecular mechanisms driving this process remain poorly defined (21). The potential pathogenic relationship between pulmonary carcinoids and SCLC remains elusive. Currently, these two tumor types are regarded as distinct and independent entities, presumed to share neither common molecular alterations nor developmental pathways. However, emerging evidence challenges this conventional dichotomy.

Recent research by Pelosi et al. suggests that at least a subset of neuroendocrine tumors exhibiting SCLC features may originate from pre-existing NETs, demonstrating shared molecular mutations between them (22). Unfortunately, these observations have not yet been validated through functional genomic experiments. Similarly, Meder L. proposed that pulmonary SCLC may manifest not only as a primary lesion or synchronous combined carcinoma, but also as a secondary tumor (23). This notion is supported by the work of Simbolo et al., who identified overlapping genetic alterations in both pulmonary carcinoids and NECs. These shared alterations include mutations in RB1, TP53, TERT, SDHA, RICTOR, PIK3CA, and MYC, albeit with differing frequencies. Cros et al. further hypothesized that NECs may evolve from carcinoids through the progressive accumulation of genetic mutations (6). While these collective findings suggest the possibility of trans-differentiation within the pulmonary neuroendocrine neoplasia spectrum, they remain largely theoretical. There have been some previous studies on “carcinoid-to-SCLC transformation” but the evidence supporting the transformation of carcinoid tumors into SCLC remains insufficient. This investigation offers a comprehensive analysis of histopathological transformation within a shared tumor spectrum. Through the integration of histomorphological transition patterns, comparative immunophenotyping, and molecular genetic data, we systematically delineate the dynamic evolution of pulmonary NEN. This approach provides robust, multidimensional validation of trans-differentiation within a clinical context. The present work addresses a significant gap in the field by empirically validating the potential for transformation from AC to SCLC, a process previously supported largely by theoretical models. In addition to documenting this phenomenon, our research aims to pinpoint the pivotal molecular drivers governing this progression. Collectively, our discoveries provide a crucial scientific basis for the future implementation of personalized precision medicine in the management of NENs.

## 2 Materials and methods

### 2.1 Clinicopathologic data

In 2023, a 60-year-old patient was admitted to our hospital for evaluation of intermittent chest tightness and dyspnea persisting for over five years, with symptomatic exacerbation occurring in the week prior to admission. Bronchoscopic examination revealed mucosal irregularity around the orifice of the left upper lobe (LUL) superior segment, along with luminal stenosis in the LUL anterior segment accompanied by an exophytic, cauliflower-like neoplasm. Histopathological analysis of the biopsy specimen confirmed small cell lung carcinoma. PET/CT imaging identified a lobulated, round soft-tissue mass (38 mm × 36 mm × 37 mm) in the LUL hilar region, demonstrating markedly increased FDG uptake, consistent with central-type lung cancer and associated obstructive atelectasis. Metastatic involvement was observed in station 5 mediastinal lymph nodes and left hilar lymph nodes. The patient received four cycles of combined immunotherapy and chemotherapy. Follow-up PET/CT revealed a significant reduction in both size and metabolic activity of the LUL hilar mass, although residual tumor viability was suspected. Correspondingly, decreased size and FDG uptake were also noted in the mediastinal and left hilar lymph nodes.

### 2.2 Hematoxylin and eosin staining

Tumor tissue specimens were fixed in 10% paraformaldehyde at 4 °C for 24 hours and processed for routine histological examination. The processing included dehydration through a graded ethanol series, clearing with xylene, and embedding in paraffin. Tissue sections were then cut, deparaffinized in xylene, and rehydrated through a graded alcohol series to water. H&E staining was performed by incubating sections with hematoxylin for 5 minutes, followed by counterstaining with eosin for 2 minutes.

### 2.3 Immunohistochemical staining

Following deparaffinization and rehydration, tissue sections underwent antigen retrieval. The sections were then incubated overnight at 4 °C with primary antibodies against TTF-1(Clone: MX011), Syn(Clone: MX038), CD56(Clone: MX039), INSM1(Clone: MXR064), RB-1(Clone:1F8), Ki-67(Clone: SP6), P53(Clone: MC008). A secondary antibody was then added to sections, followed by incubation at 25 °C for one hour. Then, wash sections repeatedly with PBS buffer, add the chromogenic reagent DAB (Lot: DAB-0031, Fuzhou Maixin biotech. Co., Ltd., China), and generate brown precipitate through enzyme-catalyzed reaction, then terminate the reaction. Observe the results under an optical microscope and take photos to record the experimental data. All antibodies were purchased from Fuzhou Maixin biotech. Co. Ltd., China.

### 2.4 DNA extraction

Total DNA was extracted from archived formalin-fixed paraffin-embedded (FFPE) samples using the QIAamp DNA FFPE Tissue Kit (Qiagen, cat. no. 56404). To maximize tumor purity, two independent pulmonary pathologists identified and marked tumor-rich regions on hematoxylin and eosin-stained FFPE sections; non-tumor components were subsequently microdissected and removed prior to DNA extraction. Germline mutations were excluded using DNA obtained from paired normal lung tissue samples. The quality of isolated genomic DNA was assessed using a combination of three methods: (1) DNA degradation and contamination were evaluated by 1% agarose gel electrophoresis; (2) DNA purity was determined using a NanoPhotometer^®^ spectrophotometer (IMPLEN, CA, USA); and (3) DNA concentration was quantified with the Qubit^®^ DNA Assay Kit on a Qubit^®^ 2.0 Fluorometer (Invitrogen, USA).

### 2.5 Whole exon sequencing

Whole-exome sequencing (WES) libraries were constructed using the Agilent SureSelect Human All Exon V6 kit (Agilent Technologies, CA, USA). Following the manufacturer’s instructions, unique index codes were assigned to each sample, and index-coded libraries were clustered on the cBot Cluster Generation System using the Illumina Hiseq PE Cluster Kit. The resulting DNA libraries were sequenced on an Illumina Hiseq platform under a paired-end 2 × 150 bp protocol. All WES procedures were performed by CapitalBio Technology Inc.

## 3 Results

### 3.1 Imaging findings

**Figure 1:**
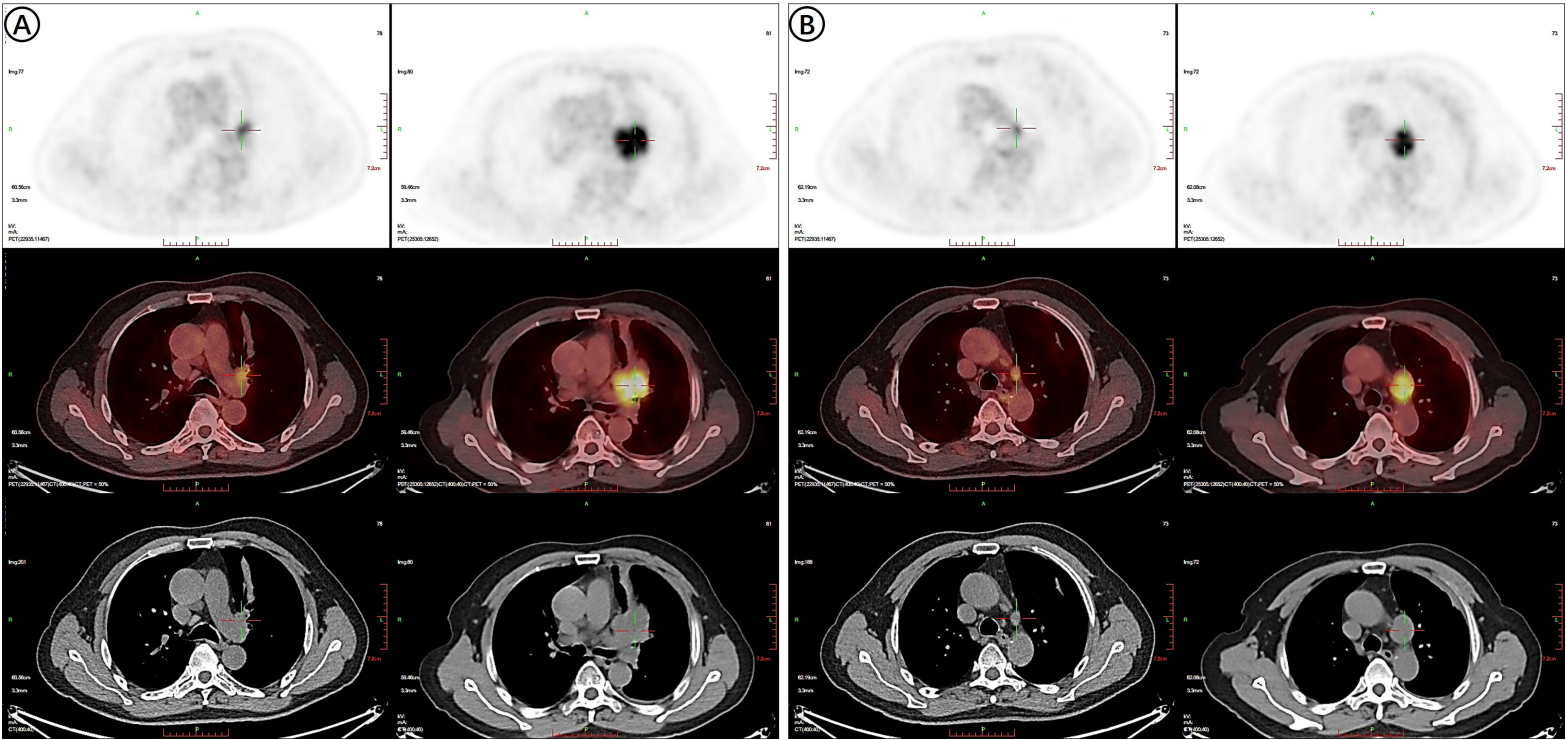
PET-CT findings of the pulmonary tumor in 2023 **(A)** and 2024 **(B)**.

Residual metabolic activity on follow-up PET-CT was consistent with persistent viable tumor. In 2023, a lobulated hilar mass (38 mm × 36 mm × 37 mm) demonstrated intense hypermetabolism (SUVmax 12.0; **Figure 1A**). After treatment, the 2024 scan showed a significant reduction in the lesion—now measuring 19 mm ×14 mm × 12 mm—with a substantially lower FDG uptake (SUVmax 4.7; **Figure 1B**).

### 3.2 Gross view of tumor

The resected left upper lobe specimen measured 20.3 × 11.8 × 3.5 cm. The bronchial resection margin had a diameter of 2.1 cm. A focal area of bronchiectasis, located 0.5 cm from the bronchial stump, was noted and was of firm consistency. Adjacent to this, the lung parenchyma was consolidated, forming an ill-defined, poorly demarcated area measuring 0.8 × 0.7 × 0.2 cm. The cut surface of this consolidated focus appeared tan to brown. The surrounding uninvolved lung tissue was tan-brown and soft in texture.

### 3.3 Histopathological morphology

**Figure 2:**
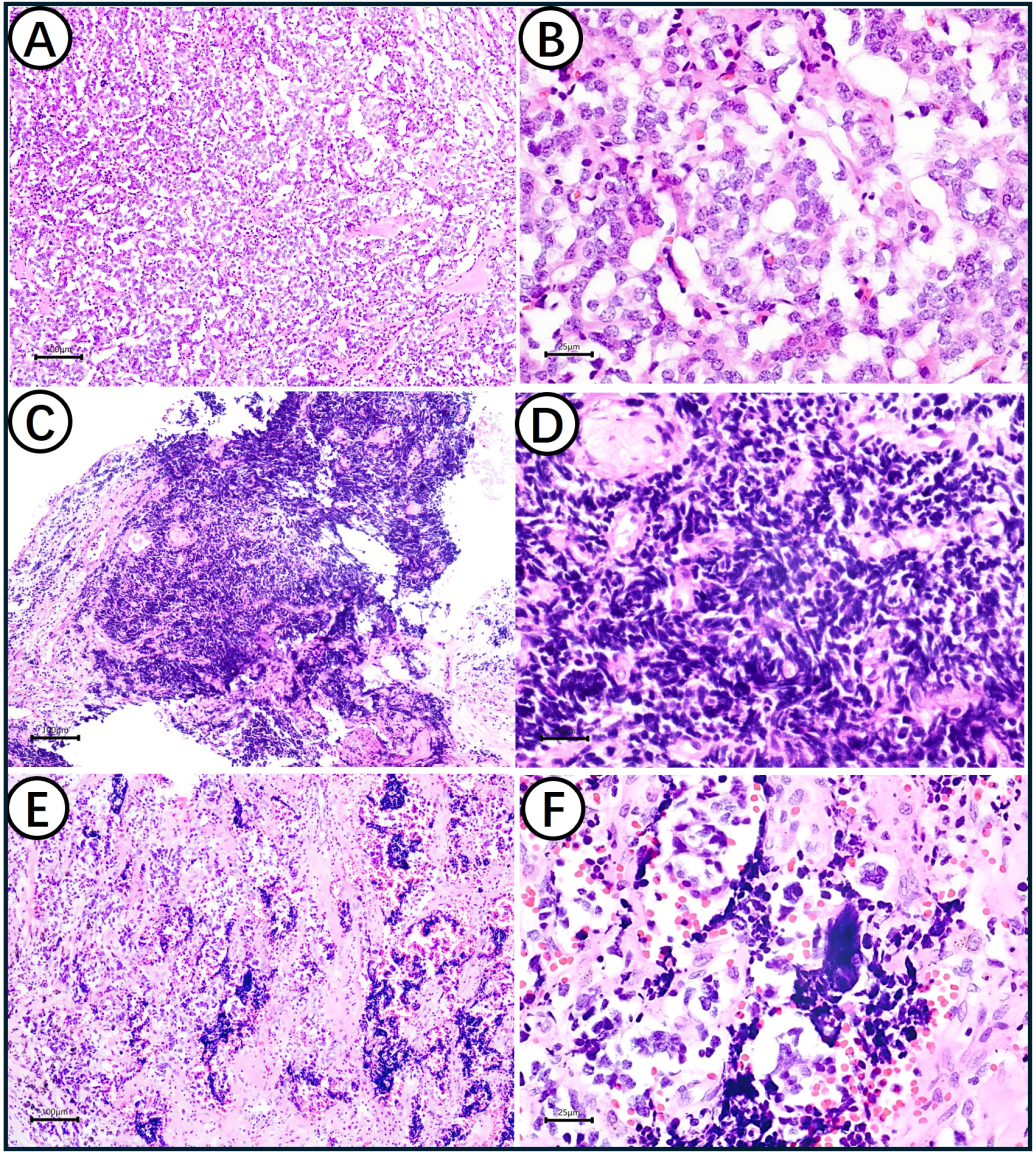
H&E staining results of AC and SCLC. **(A)** AC component present nested growth pattern, abundant pale-staining cytoplasm and focal punctate necrosis (100×); **(B)** Under high magnification, the chromatin in the nucleus of AC cells is fine and appears salt-and-pepper (400×). **(C)** SCLC has no capsule, is arranged in patches and grows invasively (100×); **(D)** SCLC component showed characteristic crushing artifact, scant cytoplasm, fine nuclear chromatin (400×); **(E)** Coexistence displaying hybrid morphological features between carcinoid and small cell carcinoma (100×). **(F)** Under high magnification, AC and SCLC are cross-distributed and fused with each other (400×E).

Post-therapy histology identified a tumor with a biphasic pattern: approximately 90% AC and 10% SCLC. The AC regions showed a nested growth pattern, abundant pale cytoplasm, and punctate necrosis (**Figures 2A, B**). The SCLC areas displayed crushing artifacts, scant cytoplasm, and fine chromatin (**Figures 2C, D**). Both components were intermingled, and transitional zones with hybrid features were identified (**Figures 2E, F**). Collectively, the coexistence, intermingling, and morphological transition between AC and SCLC provide compelling histomorphologic evidence of clonal progression from AC to SCLC.

### 3.4 Results of immunohistochemical staining

**Figure 3:**
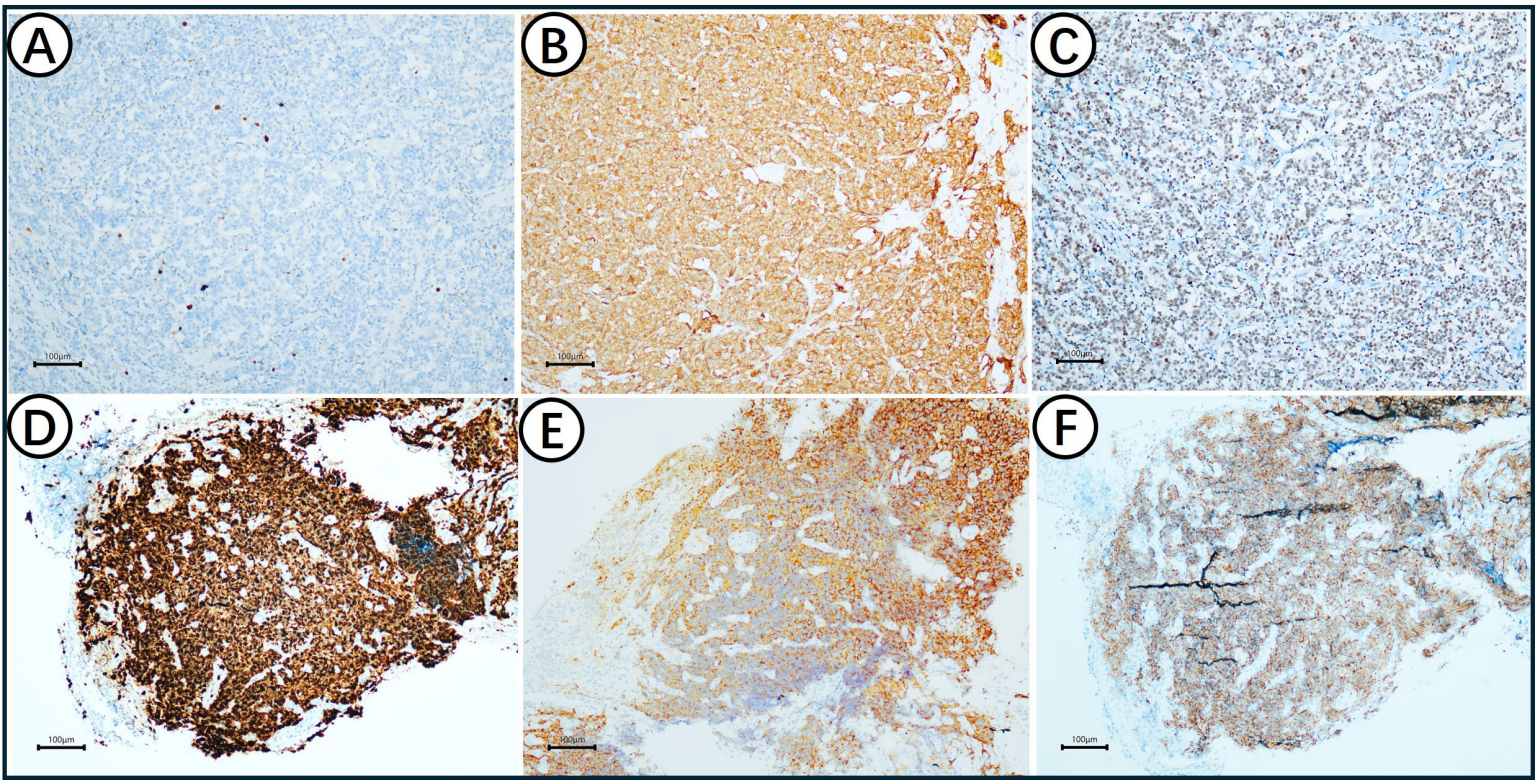
Immunohistochemical staining results in AC and SCLC. **(A)** The Ki67 index in AC is 3%; **(B)** AC expresses Syn; **(C)**AC expresses TTF-1; **(D)** The Ki67 index in SCLC is as high as 80%; **(E)** SCLC expresses Syn; **(F)** SCLC expressesTTF-1.

Immunohistochemical profiling revealed distinct proliferative indices and shared neuroendocrine markers between the two components. The AC component showed a low Ki-67 index (3%+), TP53(80%+) with loss of RB1. It was diffusely positive for the neuroendocrine markers Syn, CD56, TTF-1and INSM1 (**Figures 3A–C**). In contrast, the SCLC component exhibited a high Ki-67 index (80%+), TP53(20%+) with loss of RB1. It was diffusely positive for Syn, INSM1and TTF-1 (**Figures 3D–F**).

**Figure 4:**
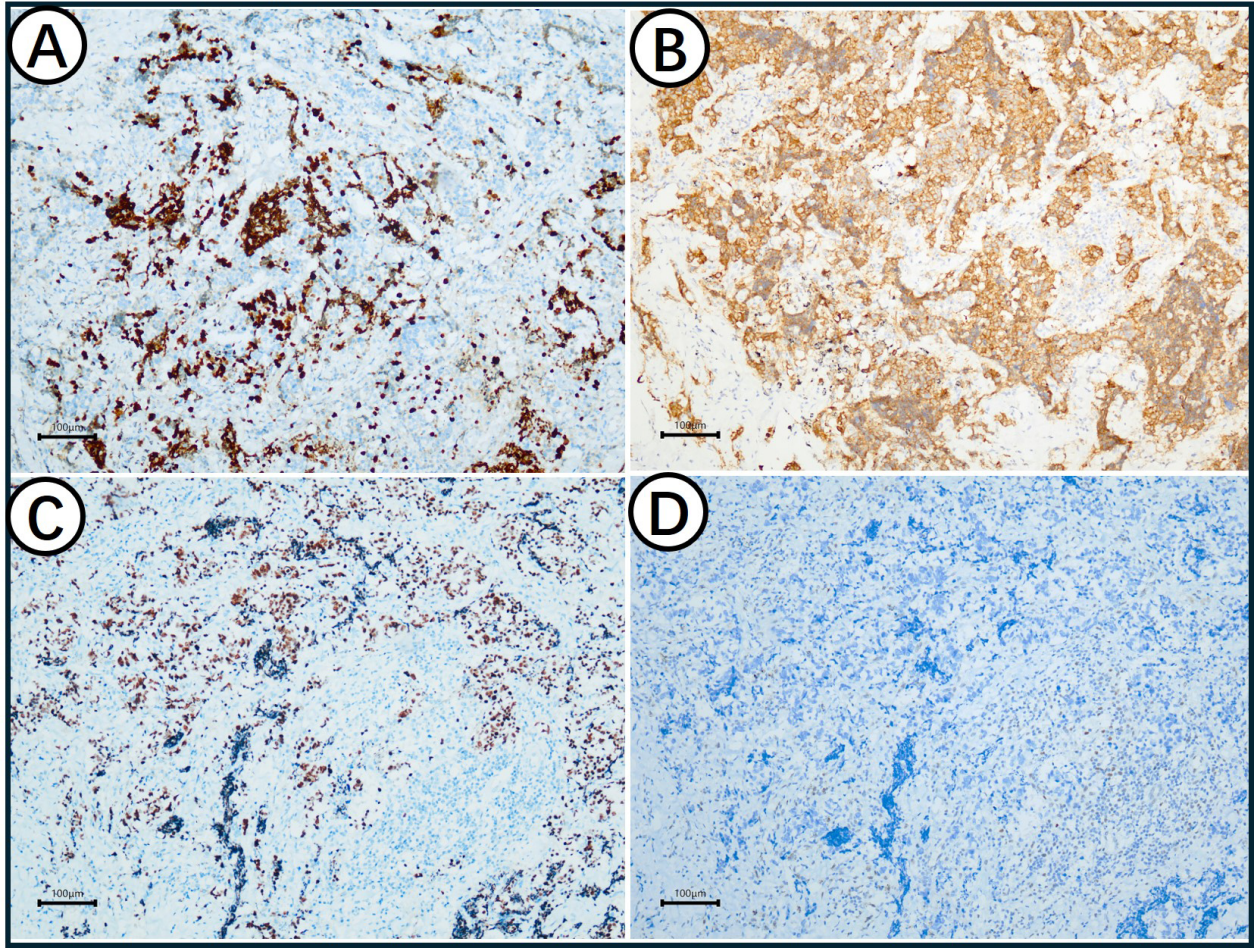
Immunohistochemical staining results in mixed region. **(A)** The Ki67 index in SCLC is as high as 80% and 3% in AC; **(B)** SCLC and AC express Syn; **(C)** SCLC expressed P53 (20%+) and AC expressed TP53(80%+); **(D)** Neither SCLC nor AC expresses RB1.

A critical immunophenotype and molecular progression was observed in the mixed region. The Ki67 index in SCLC is as high as 80% and 3% in AC (**Figure 4A**). SCLC and AC both express Syn (**Figure 4B**). SCLC expressed P53 (20%+), and AC expressed TP53(80%+) (**Figure 4C**). Neither SCLC nor AC expresses RB1 (**Figure 4D**). In terms of molecular changes, while the AC harbored an *RB1* mutation with mutant-type *TP53*, the resultant SCLC component acquired a concomitant *TP53* mutation and *RB1* mutation. This clonal evolution was further evidenced by a sharp increase in the Ki-67 index, despite both components sharing synaptophysin positivity (**Table 1**).

**Table 1:** WES identified multiple genes mutations in AC and SCLC.

Genes	Exon	Start	Basechange AA change	Mutation frequency		
				AC	SCLC	BCs
TP53	4	NM_001126115 chr17_7577094	c.C448T p.R150W	47.6%	21.8%	-
RB1	2	NM_000321.3 chr13 48881438	c.160G>T p.E54*	47.0%	23.7%	-
ARID1A	2	NM_006015 chr1_27056307	c.C1303T p.Q435X	40.2%	-	-
GNA13	4	NM_001282425 chr17_63010872	c.A352G p.I118V	38.2%	24.5%	-
CREBBP	27	NM_001079846 chr16_3786150	c.T4501A p.Y1501N	37.9%	13.8%	-
CDK6	5	NM_001145306 chr7_92300747	c.C640T p.R214C	5.0%	-	-
ABL1	11	NM_005157 chr9_133760007	c.G2330T p.R777L	4.5%	-	-
RET	2	NM_020630 chr10_43595990	c.G157A p.V53I	4.1%	-	-
MSH6	3	NM_001281492 chr2_48030588	c.C2812T p.R938X	3.6%	-	-
DDR1	10	NM_001202522 chr6_30862414	c.G1397A p.R466H	3.4%	-	-
CDKN2A	2	NM_000077 chr9_21971015	c.G343A p.V115M	3.4%	-	-
PTPRD	29	NM_001171025 chr9_8331582	c.4312_4313insT p.S1438fs	3.3%	-	-
IGF1R	10	NM_000875 chr15_99460081	c.T2177A p.L726Q	2.8%	-	-
CIC	19	NM_015125 chr19_42798840	c.G4412A p.R1471Q	2.8%	-	-
ARAF	15	NM_001256196 chrX_47430365	c.C1649A p.S550Y	2.6%	-	-
FLCN	8	NM_144606 chr17_17124769	c.C953T p.S318L	2.5%	-	-
SYK	2	NM_001135052 chr9_93606460	c.C280A p.Q94K	2.5%	-	-
TBX3	6	NM_005996 chr12_115112535	c.A1145G p.E382G	2.3%	-	-
VEGFA	1	NM_001025366 chr6_43738957	c.G514A p.A172T	2.3%	-	-
GNAQ	7	NM_002072 chr9_80336411	c.A908G p.Q303R	2.3%	-	-
RBM10	4	NM_001204467 chrX_47030644	c.C419T p.A140V	-	12.0%	-
PTPN11	5	NM_001330437 chr12_112892460	c.G618T p.L206F	-	5.2%	-
IRS2	1	NM_003749 chr13_110435635	c.C2766T p.I922I	-	3.8%	-
LYN	9	NM_001111097 chr8_56879441	c.G895A p.E299K	-	3.8%	-
PTCH1	22	NM_001354918 chr9_98209253	c.G4129A p.V1377I	-	3.5%	-
BRCA2	27	NM_000059 chr13_32972804	c.G10154A p.R3385H	-	3.4%	-
HGF	10	NM_000601 chr7_81350061	c.G1271A p.R424H	-	3.2%	-
COP1	1	NM_001001740 chr1_176175861	c.G254T p.G85V	-	3.0%	-
RICTOR	35	NM_001285440 chr5_38945155	c.C3680T p.P1227L	-	2.8%	-
KMT2D	39	NM_003482 chr12_49426484	c.C12004T p.P4002S	-	2.7%	-
RPTOR	13	NM_001163034 chr17_78831653	c.C1462T p.R488W	-	2.6%	-
ABL1	11	NM_005157 chr9_133759638	c.C1961T p.A654V	-	2.4%	-
PARP1	1	NM_001618 chr1_226595569	c.G62A p.C21Y	-	1.8%	-
GNAS	4	NM_001077489 chr20_57478737	c.C278T p.A93V	-	1.5%	-

*BCs*, *Blood controls*, referring to the patient’s peripheral blood germline DNA, used for excluding germline mutations and confirming the somatic origin of mutations detected in AC/SCLC tumor tissues.

### 3.5 Results of whole exon sequencing

WES was performed by CapitalBio Technology Inc. (Nanjing, China). Formalin-fixed paraffin-embedded (FFPE) sections of the lung tumor were subjected to comprehensive WES analysis focusing on a predefined gene panel. Sequencing data were processed using an established bioinformatics pipeline. The WES platform covers approximately 30~50 Mb of the human exome, with an effective number of potential mutation sites estimated at 3 × 10^7^ (30 Mb).

A total of 1000 somatic mutations were detected in the AC component, and 1015 were identified in the SCLC component. Among these, 160 mutations—located at identical genomic positions in the same genes—were shared between the two tumors. This shared mutation set accounted for approximately 16% of the total mutations in each tumor component, a proportion substantially higher than would be expected by chance between two independent tumors (see formula for statistical validation).


$$E[S]=\frac{1015\text{ x }1000}{3\text{ x }10^{7}}=\frac{1,015,00}{30,000,000}\approx 0.0338$$

Under the null hypothesis of independent tumor origins, the expected number of shared mutations was 0.0338. The observation of 160 shared mutations allows us to decisively reject this hypothesis, as the observed value vastly exceeds the expectation (160 >> 0.0338), thereby strongly supporting a common clonal origin.

In addition to the shared mutations, we further analyzed the unique genetic alterations in the SCLC component to explore the molecular mechanisms underlying AC-to-SCLC transformation. The SCLC component exhibited specific mutations not detected in AC, including RBM10 (12.0%), PTPN11 (5.2%), IRS2 (3.8%), LYN (3.8%), and others. These unique mutations may be involved in the malignant progression of SCLC, as RBM10 and PTPN11 have been previously implicated in the regulation of tumor cell proliferation and differentiation in lung cancer (24, 25). These SCLC-specific mutations are likely secondary events acquired during AC-to-SCLC transformation, potentially contributing to SCLC’s highly aggressive, poorly differentiated phenotype.

### 3.6 Clonal evolution analysis

**Figure 5:**
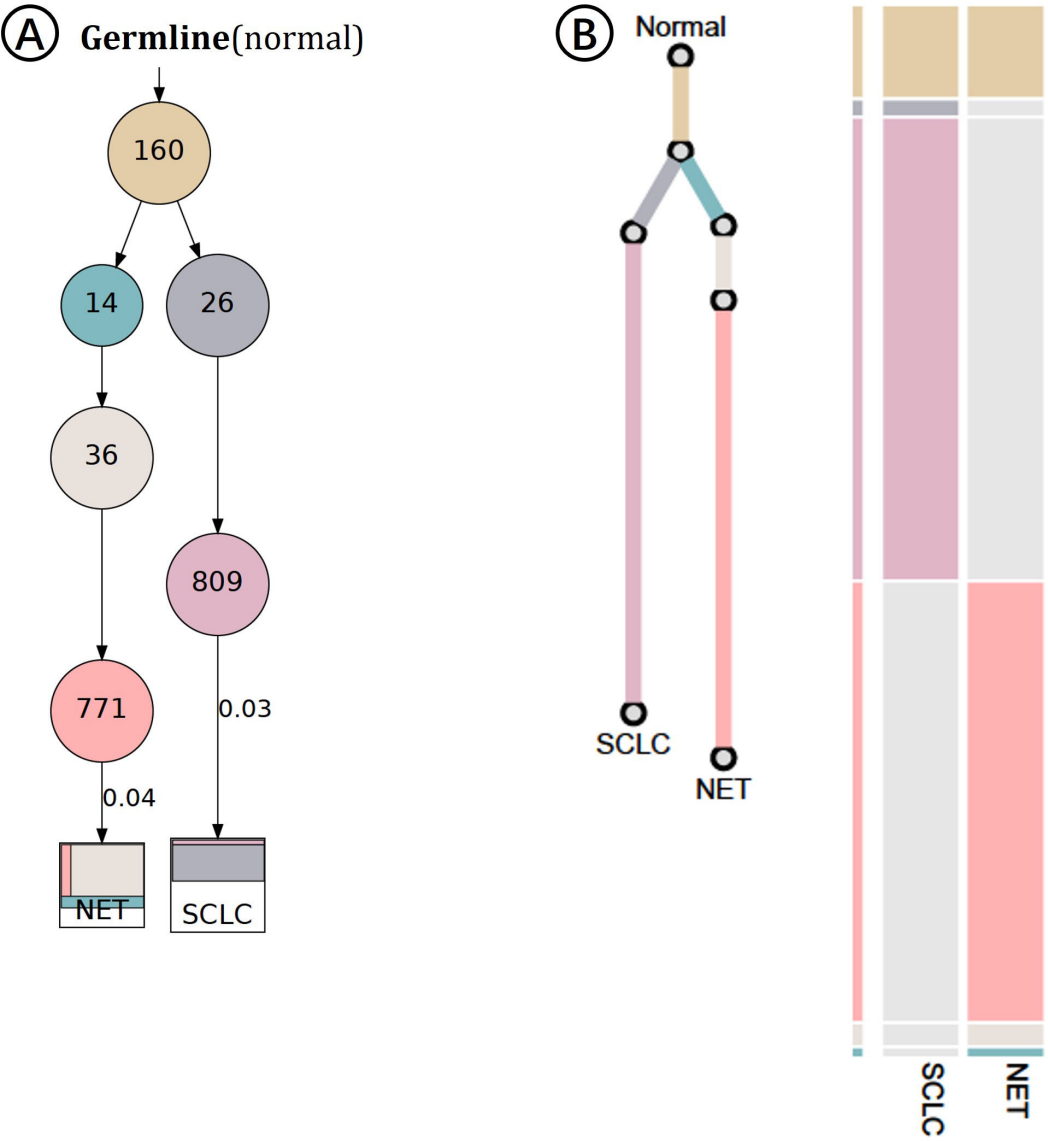
The results of clonal evolution analysis support a “branching evolution” model.

Clonal evolution analysis confirmed that the AC and SCLC components shared identical major driver mutations, indicating a common ancestral clone. The tumor cell content was estimated at 95% in the AC and 70% in the SCLC. The divergent *TP53/RB1* status between the two components suggests a model of branched evolution. In this model, one lineage (giving rise to the AC) remained genetically stable, while the other (giving rise to the SCLC) acquired profound genomic instability, ultimately driving its transformation into a more aggressive and poorly differentiated phenotype. 0.04 and 0.03 respectively represent the average VAF values of clone 771 and clone 809 (**Figure 5**). We objectively addressed the potential impact of neoadjuvant immunochemotherapy on tumor evolution, and the clinical timeline of this case fundamentally excludes therapy-induced AC-to-SCLC transformation: the tumor was initially diagnosed as SCLC, and the AC component was only identified in the surgical specimen after 4 cycles of neoadjuvant therapy—this is the reverse of the timeline required for therapy-induced transformation from AC to SCLC. The coexistence of AC and SCLC is best explained by the two components being pre-existing clonal branches from a common ancestral clone prior to treatment; neoadjuvant immunochemotherapy did not induce clonal transformation, but rather altered the tumor’s clonal fraction, suppressing the originally dominant SCLC clone and unmasking the occult AC clone that was undetectable at initial diagnosis.

## 4 Discussion

NENs constitute a heterogeneous group of tumors with diverse clinical manifestations and biological behaviors. Current epidemiological data indicate that pulmonary carcinoids occur predominantly in younger populations, with incidence rates showing a consistent linear increase over time (26). Carcinoids are histopathologically defined as well-differentiated NENs. They are characterized by low mitotic activity (<10/2 mm²), absent or focal necrosis, moderate to abundant cytoplasm, fine “salt-and-pepper” chromatin, and strong expression of neuroendocrine markers including Syn and CgA (27). At the molecular level, these tumors frequently harbor mutations in genes involved in chromatin remodeling, including *MEN1, PSIP1, EIF1AX*, and *ARID1A*, which correlate with their generally favorable prognosis (28, 29). In contrast, SCLC presents a starkly different clinicopathological profile. SCLC is characterized by high-grade histologic features consistent with marked proliferative activity. These include tumor cells with marked crowding and nuclear molding, fine chromatin, inconspicuous nucleoli, scant cytoplasm, and frequent pathological mitotic figures. Genomically, SCLC demonstrates recurrent alterations in *TP53, RB1, KRAS*, and *STK11/KEAP1*. To date, no curative systemic therapy exists for advanced SCLC, contributing to its aggressive clinical course and poor prognosis (30).

AC and SCLC exhibit profound differences in molecular profiles, treatment strategies, and survival outcomes. Therefore, investigating the potential for AC-to-SCLC transformation is a critical area of research, with significant implications for precision medicine and patient management. The prevailing pathological paradigm regards AC and SCLC as biologically distinct entities with no established pathogenic continuum (31–33). However, emerging evidence challenges this conventional view. Pelosi et al. have suggested that a subset of SCLCs may originate from pre-existing neuroendocrine tumors (22). Similarly, Simbolo et al. reported overlapping genetic alterations in AC and SCLC (8), while Cros et al. proposed that SCLC may evolve from AC through the stepwise accumulation of genetic mutations (6). Meder et al. further delineated two putative oncogenic pathways for SCLC. The first involves primary tumorigenesis from neuroendocrine stem cells with biallelic TP53/RB1 loss. The second describes secondary SCLC arising from NOTCH-deficient precursors, which acquire *RB1* inactivation on a background of pre-existing *TP53* mutation (23). Although these studies collectively suggest the possibility of trans-differentiation within the pulmonary NEN spectrum, they remain largely theoretical and lack direct validation in clinical patient cohorts. Whether carcinoid can transform into SCLC requires more conclusive evidence for confirmation.

In this study, histopathological examination revealed an intermingled coexistence of AC and SCLC components without clear demarcation, forming morphologically transitional zones and effectively excluding a collision tumor. Immunohistochemical findings combined with WES results, which significantly exceeded expectations for independently arising tumors, strongly support a common clonal origin. Analysis of somatic SNVs and CNVs further confirmed their derivation from a common progenitor cell. These integrated findings provide robust evidence for a “branching evolution” model, indicating early clonal transformation from AC to SCLC.

Surgical resection is not a conventional first-line treatment for SCLC. Current guidelines recommend chemotherapy or chemoimmunotherapy as the standard initial regimen for limited-stage SCLC. However, this patient presented with stage IIIA (cT2N2M0) disease. After four cycles of neoadjuvant chemo-immunotherapy, PET/CT demonstrated significant shrinkage and metabolic reduction in both the primary lesion and lymph nodes. This response achieved a deep clinical remission with downstaging. The multidisciplinary team assessment concluded that an R0 resection was feasible. The patient had an Eastern Cooperative Oncology Group performance status of 0 and no cardiopulmonary comorbidities. Surgical tolerance was assessed as good. Following thorough informed consent, the patient maintained a strong preference for surgical intervention. Current evidence suggests that neoadjuvant chemoimmunotherapy for resectable SCLC leads to a higher pathological complete response rate and demonstrates superior efficacy compared to chemotherapy alone (34). In this study, as of the last follow-up on January 16, 2026, no tumor recurrence or metastasis was observed. The overall survival (OS) follow-up duration was 24.7 months, and the postoperative progression-free survival (PFS) was 19.9 months. Neither OS nor PFS endpoints were reached. These survival durations have exceeded the median OS (18–24 months) for stage IIIA SCLC treated with conventional chemoradiotherapy combined with immunotherapy.

This study integrates multilevel evidence—including histomorphology, immunohistochemistry, and molecular profiling from a real-world clinical case—to support the possibility of AC transforming into SCLC. This finding helps address a gap in current knowledge, which has until now been largely theoretical. In the present case, the residual tumor within the tumor bed was predominantly composed of AC, with a minor component exhibiting features of pulmonary SCLC. Both components were located within the same tumor bed and showed areas of intermingled growth. We propose that the SCLC component likely emerged through progression from the AC. Notably, the SCLC component demonstrated sensitivity to the current treatment regimen, whereas the AC component appeared relatively less responsive.

Our study has several limitations. It arose from the serendipitous identification of coexisting carcinoid and SCLC components within a single pulmonary lesion, which is inherently limited by its single-case nature. The generalizability of our findings to all pulmonary atypical carcinoid patients is constrained, as this case may represent a rare subset of AC with specific molecular features predisposing to SCLC transformation. To address this limitation, future research should focus on conducting multi-center collaborative studies and large-cohort analyses of AC-SCLC mixed tumors to verify the universality of AC-to-SCLC transformation and identify potential high-risk subgroups of AC patients. Most prior studies regard carcinoids and SCLCs as distinct and independent tumors. However, our observations indicate that these two tumor types can intermingle, suggesting a potential biological relationship between them. To investigate a potential common clonal origin, we analyzed molecular genetic alterations, progressive genomic abnormalities, and phenotypic transitions in these tumors. This work aimed to validate the hypothesis of carcinoid-to-SCLC transformation. Although the limited number of cases may restrict generalizability, our data suggest that at least a subset of carcinoids may harbor the potential for such transformation. These findings carry important clinical implications, indicating that early intervention in patients with carcinoids might prevent progression to SCLC. However, our study could not determine whether this transformation is driven primarily by accumulated gene mutations or by epigenetic reprogramming. Further research is needed to clarify the precise molecular drivers, which may provide a theoretical foundation for personalized precision therapy in this patient population.

## 5 Conclusion

This study provides a comprehensive investigation into histopathological transformation within the same tumor spectrum. Through a multidimensional analysis of clinical, histopathological, and molecular data from actual cases, we offer direct evidence demonstrating the dynamic progression of NENs. Specifically, our findings confirm the potential for transformation from AC to SCLC.

As emphasized, a key future direction is the functional validation of key mutations identified in this study. These include the common core mutations (*TP53, RB1*) and SCLC-specific mutations (RBM10, PTPN11). Their roles in the transition from carcinoid to SCLC are not fully understood. Functional experiments, such as *in vitro* and *in vivo* models, could be conducted to clarify their pathogenic mechanisms. Furthermore, large-scale, multicenter studies are needed to discover more similar cases. This would help verify the generalizability of the carcinoid-to-SCLC transformation. These efforts will facilitate the translation of our findings into clinical practice. They will also advance the management of pulmonary neuroendocrine tumors.

### 5.1 Key points of clinical practice section

Our findings address a critical gap in the current theoretical framework by delivering concrete pathological and molecular support, thereby establishing a new foundation for personalized precision medicine in the management of NENs.

## Data Availability

The datasets presented in this study can be found in online repositories. The names of the repository/repositories and accession number(s) can be found in the article/supplementary material.
